# Turkish students’ perceptions of professionalism at the beginning and at the end of medical education: a cross-sectional qualitative study

**DOI:** 10.3402/meo.v20.26614

**Published:** 2015-03-19

**Authors:** Mustafa Volkan Kavas, Meral Demirören, Ayşen Melek Aytuğ Koşan, Süleyman Tuna Karahan, Neyyire Yasemin Yalim

**Affiliations:** 1 Department of Medical History and Ethics, Faculty of Medicine, Ankara University, Ankara, Turkey; 2 Department of Medical Education and Informatics, Faculty of Medicine, Ankara University, Ankara, Turkey; 3 Department of Medical Education and Informatics, Faculty of Medicine, Selçuk University, Konya, Turkey; 4 Department of Anatomy, Faculty of Medicine, Ankara University, Ankara, Turkey

**Keywords:** professional identity development, professionalism, medical education, hidden curriculum, role models

## Abstract

**Aim:**

Medical students’ perceptions of professionalism might reflect the impact of the current educational processes on their professional identity development. This study focuses on Ankara University Faculty of Medicine students’ perceptions of ‘good doctor’ along with the factors effective on the formation of these perceptions.

**Method:**

Six focus groups with 59 medical students from Grade-1 and Grade-6 were held. The transcripts of discussions were analyzed thematically.

**Results:**

Results regarding ‘being a good physician’ mostly mirrored the findings of previous studies framing the medical professionalism concept. The thematic pattern of the discussions on the relation between professional development and medical education suggests that students suffer from a gradual *erosion of perception* during medical education. That *the education cannot either change the person for the better or might downgrade the person instead of improving her/him* were shared by participants from both grades. Students consider *clinical practice* and *role models* two main variables determining the person's qualification as a professional.

**Conclusions:**

The formal and hidden programs determine the quality and efficacy of the professional education together. Attempts to restructure medical education must recognize the reciprocal dynamics between these two components and, thus, should carefully work out the practical aspect of the educational processes.

Medical professionalism has been deliberated for more than a decade by ethicists and medical educators (1–3). It is a multidimensional and value-laden phenomenon, involving many social, historical, institutional, and contextual elements (4, 5). Although debates continue on the scope and function of medical professionalism, it is seen today as one of the core competency domains in medical education (6–11).

Many scholars argue that teaching professionalism is not possible solely via formal education programs (12, 13), but could be achieved more effectively by the informal, ‘hidden curriculum’ (14, 15). In particular, the quality of role models may have a profound impact on the development of students’ professional values (16–19). Furthermore, common social understandings, medical culture, manners of clinical communication, and the organization of the healthcare setting have a major influence on the development of medical professionalism (20, 21).

Medical students’ perceptions of professionalism may reflect whether the current education process promotes professional values and behaviors. This study, then, focuses on Ankara University Faculty of Medicine (AUFM) students’ perceptions of ‘a good doctor’ –providing insights into what are considered desirable traits and how these can be acquired. Structured educational activities concerning professionalism are considerably limited in AUFM, which may highlight shortcomings in relying largely on informal, ‘hidden’ curricula. It is hoped that the resulting data will help to improve students’ professional development in AUFM and beyond.

## Method

### Design

This study evaluated AUFM students’ perceptions concerning ‘being a good physician’ along with the factors effective on the formation of these perceptions. The term ‘being a good physician’ was preferred in this study, since it covers the rich semantic content of the ‘professionalism’ concept in Turkish, and thus, is more readily understood by students. The study utilized a cross-sectional design, with qualitative data emerging from focus groups with medical students.

### Working group

AUFM Grade-1 (G1) and Grade-6 (G6) students were recruited.

At AUFM, the formal education program of G1 consists largely of a series of large, classroom lectures and laboratory practices called ‘Knowledge of Structure and Function’ – although students also attend small group practices for basic technical skills education (22). In addition, they take a 10-week introductory course on ‘professional identity’ composed of seminars, small group discussions, film sessions, and museum visits.

G6 students, on the other hand, work in the clinic for one whole year – rotating through various clinics and spending 1–2 months in each department. They are seen as members of the healthcare team, and are responsible for much of the basic medical care provided – such as taking anamnesis, physical examination and follow-up of patients, polyclinic services, night duties, etc. Of course, they must obey the orders of superiors, follow hospital rules, and adhere to specified working hours. They are also expected to attend clinic meetings, seminars, and literature presentations (22).

Focus groups were conducted with G1 students at the end of the first semester and with G6 at the beginning of the second semester. Although G1 students were still ‘freshmen’, G6 students had only several months before graduation.

G1 students were included because they have little or no medical training – allowing examination of their moral preconceptions, motivations regarding their professional futures, and expectations from their ongoing medical education. In contrast, G6 students have completed basic and clinical education programs as well as considerable practical experience as interns. By focusing on opposite ends of the educational spectrum, we sought to examine the extent to which students’ perceptions were shaped by direct observations and first-hand experiences – as well as provide insight into their moral environment.

In total, six focus groups with participants from both grades (three groups for each) were conducted. In the 2012–2013 academic year, there were 313 (164 female, 149 male) G1 students and 263 (142 female, 121 male) G6 students. A systematic random sample of 24 was drawn from each grade, stratified by sex (G1) and internship location (internal diseases vs. surgery) for G6. In total, 59 students (31 G1, 28 G6) participated in the focus groups, which ranged in size from 8 to 12 participants. Females comprised slightly more than half (54%) of the sample. The study protocol was approved by the AUFM Clinical Research Ethics Committee, and written and oral consent was garnered from all participants.

Group sessions were guided in a semi-structured format by the following questions (key items):

1. What comes to your mind when I say ‘to be a good physician’?
2. What do you think are the necessary features a person must have in order to practice as a ‘good physician’?
3. Can you tell a behavior which could be an example to the practice of ‘a good physician’?
4. Have you ever witnessed a behavior unfit for a ‘good physician’?
5. Do you think being a ‘good physician’ can be learned? How?
6. If you ever had a chance, what advice would you give to the institutional administrators so that they can raise good physicians?

### Analysis of focus group discussions

The transcripts of the group discussions were subjected to the thematic analysis as outlined in Table 1. First, each transcript was read independently by two researchers and compared with the audio recordings, when necessary. The first drafts were then revised and technically corrected, allowing the researchers to develop an initial understanding of the data (Step 1: Familiarization). Transcripts were then reread to extract and group relevant answers to the key questions. Accounts which could not be classified or were deemed to relate to multiple questions were situated under another cluster to be reviewed separately. Each researcher applied this protocol operation on the discussion log for which they were responsible (Step 2: Data Deconstruction). Subsequently, moderators (group facilitators and assistants) cross-checked their classifications and assigned themes to these groups (Step 3: Thematic Identification). While working on the thematic coding, researchers subjectively assessed the *frequency* (how frequently something was said, though not counted), *specificity* (richness in terms of information and detail), *emotional content* (passion, enthusiasm, or intensity) and *extensiveness* (how many people said something) of the accounts (23), p. 136). Consensus meetings of all researchers were then held to discuss and agree on the respective theme set (Step 4: Indexing) – with contexts, themes, and sub-themes subsequently organized into tables (Step 5: Charting). The researchers then reevaluated their initial analysis on the basis of these tables and reconstructed the data, defining the pattern of relations between contexts, themes, and sub-themes (Step 6: Mapping). Samples of extracted accounts which best represented each of the themes or sub-themes were selected, some of which were excerpted with approval of the researcher who transcribed the session. Two researchers interpreted the final results with respect to the relevant literature and the current medical training at AUFM (Step 7: Interpretation). Finally, the draft report was revised after consultation with the rest of the research group.

**Table 1:** Analysis steps

Stage	Step	Function	Aim
Comprehensive understanding/recontextualization	7	Interpretation	Reaching an understanding and insight
Structural analysis/decontextualization	6	Mapping	Reconstruction of the data: Defining the pattern of relations between contexts, themes and sub-themes
	5	Charting	Creating tables for contexts, themes and sub-themes
	4	Indexing	Debate and consensus on the themes set
	3	Cross check of the defined themes	Identification of a thematic framework
	2	Deconstruction of the raw data	Extracting relevant answers to questions
Naïve understanding	1	Rough reading	Familiarization

## Results

### Connotations about ‘being a physician’

Participants’ accounts exposed three interrelated features framing their perceptions of ‘being a physician’: First, students addressed the issue primarily on the individual level, while rarely touching upon its societal aspects. Second, students often drew no distinction between traits attributed to ‘being a good person’ and ‘being a good professional’. Last, connotations of ‘being a physician’ were predominantly positive; in fact, G1 students had no negative views in this regard. Additionally, G1 students offered stronger and more diverse opinions than their G6 counterparts. Conversely, G6 students offered negative associations related to clinical overwork (e.g., sleeplessness, weariness) and violence towards physicians (Table 2).

**Table 2:** The thematic pattern of the students’ expressions concerning connotations of ‘being a physician’

Connotations about ‘being a physician’– The thematic pattern				

			Theme	
			
+/−	Level	Dimension	Grade-1	Grade-6
Positive	Individual	Being a good person	Having sedateness Being altruistic Being genial Being merciful Showing empathy	Having sedateness Being altruistic
		Being a good professional	Being experienced Being knowledgeable and skillful Being trustworthy Being coldblooded Being responsible Giving people hope Having communication skills Saving lives	Being experienced Being knowledgeable and skillful Healing people
	Societal		Profession as a special means of communication with people Improving the quality of life of people	Profession involving a one-to-one communication
Negative	Individual		–	Weariness Sleeplessness
	Societal		–	Being exposed to violence

### Perceptions of ‘being a good physician’

Although themes related to ‘being a good physician’ could be classified as ‘being a good person’ or ‘being a good professional’, limited data precluded evaluation of the former.

#### Being a good professional

Most student accounts involved qualities, attitudes, and behaviors they perceived as necessary features of *physician identity* which, in turn, were directly grounded in some distinctive contexts: 1) professional responsibility; 2) physician-patient relationships; 3) physician-physician/healthcare professional relationships; and 4) responsibility of the physician as an intellectual (Table 3).

**Table 3:** The components of the dimension of ‘being a good professional’ and behaviors unfit for ‘a good physician’ extracted from students’ accounts–themes/sub-themes/samples

		Being a good professional		Behaviors unfit for a ‘good physician’	
			
		Sub-theme		Samples	
			
Context	Theme	Grade-1	Grade-6	Grade-1	Grade-6
Professional responsibility	Relying on scientific knowledge	Doing the right thing scientifically	Doing the right thing scientifically	–	–
	Competency	Being knowledgeable and skillful	Being knowledgeable and skillful Being able to update knowledge	‘Being incompetent in terms of knowledge and skills’	–
	Accuracy	–	Attentive conduct of the profession Giving the right treatment Making an accurate diagnosis	‘Giving unnecessary treatments’ ‘Jeopardizing the patient's life with malpractice’ ‘Ordering unnecessary tests’	‘Being late for consultations or not showing up at all’ ‘Delaying the tests’ ‘Medical errors or negligence because of lack of knowledge and/or insensitivity towards patients’
	Objectivity	Acting unbiased	Acting unbiased	‘Not treating patients equally’ ‘Acting unbiased’ ‘Changing her/his behavior according to the persons’ appearance’	‘Not treating patients equally’ ‘Favoring private patients’ ‘Tending to treat patients badly because of their worldviews’
	Being principled	Keeping financial interests in the background while conducting the profession	Prioritizing the professional responsibilities	‘Performing unnecessary medical interventions to acquire unfair profit’ ‘Viewing medical professional just a means for making money’ ‘Viewing patients as customers’	‘Reflecting her/his own personal matters onto her/his professional life’ ‘Regardless of the expected benefit, giving treatment/prescribing drugs to please the patient’
	Being able to work under pressure	Being able to think fast Being able to make decisions under pressure Cold-bloodedness	Being able to think fast	–	–
	Being a researcher	–	Making research	–	–
	Being an educator	–	–	–	‘Not being a good educator’ ‘Not sparing time for educational duties’
Physician-patient relationship	Empathy	Establishing empathy with patients Being a good listener Sensitiveness to patients’ suffering	Establishing empathy with patients Being interested in patients Understanding patients	‘Being indifferent to the patient's suffering’	‘Being insensitive while breaking bad news’
	Trustworthiness	Reliability Not viewing patients as customers	Being accessible	‘Behaving indifferent towards the patient’ ‘Insensitivity’ ‘Reflecting her/his own personal concerns onto her/his relationship with the patient’ ‘Viewing patients as customers’	‘Being inaccessible’ ‘Not being close to the patient’
	Communication	Being comprehensible/understandable Informing patients Having communication skills Being promising/giving hope Debonairness Relieving patients	Being comprehensible/understandable Informing patients/their relatives Having communication skills	‘Behaving rude/inattentively towards patients/their relatives/third parties’ ‘Having a high ego’ ‘Looking down upon others in her/his relationship with patients’	‘Not providing the patient with adequate information/not informing the patient’ ‘Not touching the patient’ ‘Misbehaving towards the patient’ ‘Being angry and/or straight-faced’ ‘Not listening to the patient’
	Respect for patients’ autonomy	Showing a caring attitude Acting without prejudice in the relationship with patients	Showing a caring attitude	–	‘Using a language disrespectful to the patient's personality’. (e.g.,: ‘shooting the patient’, ‘pickle patient’) ‘Taking care of the patient as a human being, not just the illness’
	Fidelity	–	Following up the patient Not letting the patient down	‘Neglecting the patient’	–
	Holistic treatment	–	Evaluating the patient in relation with her/his environment	–	–
	Respect for patients’ privacy	–	–	–	‘Being disrespectful towards patients’ privacy’
Physician-physician/healthcare professional relationship	Teamwork	Being able to work as a part of a team Having good relationships with colleagues	Being able to work as a part of a team Having good relationships with colleagues	–	Abusing professional hierarchy (‘assigning subordinates with heavy workload’)
	Competition	–	Being aware of her/his responsibilities towards colleagues	–	‘Competing with colleagues’
Responsibility of the physician as an intellectual	Openness	Having flexible value judgments	–	–	–
	Being active	Being socially active	–	–	–
	Leadership	Being a model for people around	–	–	–
	Having opinions on paramedical issues	Being lettered	–	–	–

#### 1) Professional responsibility

All groups contended that good physicians should have basic medical knowledge and relevant professional skills. They should behave calmly and decisively under pressure; rapidly solve problems on the ward; be careful while conducting diagnostic and treatment procedures; develop and offer fair approaches to peers, subordinates, and patients; and choose the right medical interventions for their patients. Participants also recognized the importance of continually developing clinical knowledge, communication skills, and team-working skills to help improve standards.

In addition, according to students from both grades, physicians should neither discriminate nor give unfair priority to patients. The majority of the G6 students thought that physicians should prioritize their professional responsibilities over the pursuit of their own interests. Similarly, G1 students mentioned that physicians should not put financial interests before professional values.

Recently I and a friend from class saw a car, while chatting. ‘Wow, I will buy one of these when I become a doc’ he said. And I asked ‘you think you'll earn that much, which specialty are you into?’ He looked at me weirdly and said ‘don't you dare to say that you came here to help people.’ (FG-5/Part.3/Line 919–922) (G1) One should not regard the patient as a customer. I mean, when you go to a private hospital, they require some tests, some irrelevant things from you …. They do all sorts of things apart from your illness. They call it ‘the general overview’ but the patient is treated like a fool especially for making money out of such things. (FG-4/Part.8/Line 285–290) (G1)

#### 2) Physician-patient relationship

Participants from both grades mentioned that good physicians should have adequate time for their patients, approach and treat them with care and patience, and care for them as persons rather than objects. Sub-themes were classified as follows: empathy, trustworthiness, communication, respect for patient autonomy, fidelity, and holistic treatment. ‘Establishing empathy with patients’ and ‘having communication skills’ provoked the most extensive and intensive discussion. Interestingly, G1 students almost never touched on the issues of fidelity and holistic treatment (Table 3).

G1 and G6 students also differed on physicians’ approaches to patients. Some participants claimed that the patient should be treated in a friendly manner, whereas others considered that ‘pleasing the patient’ is not a priority of a good physician. G1 students emphasized the ‘communication’ aspect of the physician-patient relationship. However, G6 students emphasized that physicians knows best and should utilize their competency, knowledge, and skills in patients’ best interests.

… we are not going to be engineers my friends, you know, people who'll come to us have feelings too. Where does the vein lead, I mean, what results occur when I intervene with which vein, I must know all of it. A machine engineer might know everything about a machine as well; but we aren't going to be machine engineers. Because, you can behave shrewishly to a machine, you don't need to smile, but with a person you have to be in a friendly manner definitely. (FG-6/Part.10/Line 168–175) (G1)

#### 3) Physician-physician/healthcare professional relationship

Both groups referred to the place of the physician within a professional healthcare team and to the tasks and responsibilities arising from this position. Relatedly, students felt that physicians should have a responsibility towards their patients, the collective team, and individuals and institutions with which the team interacts. Furthermore, although ‘competition among healthcare professionals’ was never mentioned by G1 students, it was frequently expressed (usually negatively) by G6 students.

#### 4) Responsibility of the physician as an intellectual

All accounts relative to this aspect of professionalism came from G1 students – who suggested that physicians should be socially active, intellectually competent, open-minded, and good role models.

#### Behaviors unfit for ‘a good physician’

Students had difficulty contributing narrations of ‘good practice’, so it was not possible to differentiate between ‘features attributed to being a good physician’ and ‘behaviors/actions of a good doctor’. However, they were able to give clearer, more specific examples of behaviors unfit for a good physician.

G1 students’ examples centered largely on physician-patient encounters they had witnessed as a patient or as a patient's relative, whereas G6 students shared experiences they had observed as interns in the clinical environment.

Because the doctor was so much praised, I went to him …. He said ‘we met before.’ However, it was my first time there …. I had a problem in my eye. I asked ‘would it be because of this and that?’ He said ‘maybe’ or so. I mean, he never addressed comments to me; he didn't even speak with me. (FG-4/Part.8/Line 662–669) (G1)

The reason why patients go from door to door is that we don't explain their illnesses completely. I mean we give a drug, as if it would heal them. Perhaps it might do them better but in some illnesses, it can't ease it completely, but patients do expect that it would. Because we don't explain it completely, they view us as bad doctors and then go from door to door. (FG-1/Part.2/Line 133–137) (G6)

The analysis revealed that participants’ experiences were based predominantly on ‘professional responsibility’ and ‘physician-patient relationship’ (Table 3). In contrast, the ‘responsibility of the physician as an intellectual’, previously mentioned by G1 students, was almost never referred to by either group. In addition, G6 students brought forth the sub-theme ‘being a good educator’– suggesting that some physicians fell short in this responsibility. In particular, negative experiences regarding students’ communication with their teachers and senior colleagues (e.g., residents) were highlighted. Moreover, some of the G6 students’ accounts suggested that they had frequently witnessed a clinical disregard of ‘respect for patient autonomy’. Last, examples by G1 students related to ‘keeping financial interests in the background while conducting the profession’ were not expressed by the G6 groups.

### ‘Being a good physician’ and medical education

Students’ perspectives on roles of medical education in becoming ‘good physicians’ were discussed.

#### The possibility of becoming ‘good physicians’ through education

Participants’ answers to the question of whether or not becoming a ‘good physician’ can be taught and learned suggest a trilateral thematic pattern.

The idea that ‘being a good physician’ cannot be taught through specific education programs, or can be taught only if the learners’ personality is amenable to such approaches, was notably expressed by G1 students. Comments from G6 students, in contrast, were more intense and negative. According to them, the present education system produces ‘bad physicians’, and the belief that the essential elements entailed in becoming a ‘good physician’ can be conveyed via education was rarely articulated by participants from either grade.

Those who believed that education could have a positive effect considered it a conditional process. That is, variables ‘intrinsic to the individual’ and variables ‘extrinsic to the individual’ were seen as neither complementary nor interrelated – but as *independent determinants*. On several occasions, students implied that these two groups of variables are of equivalent necessity. However, most students expressed that both individuals’ inner desire and capacity to learn are necessary to facilitate learning. Among the second group of factors, ‘the presence of influential role models’ and ‘the determinative effect of praxis in learning processes’ were predominant.

They assigned us to working with a resident in the internal diseases out-patient clinic … In that month, whatever I saw from her, I mean in the out-patient clinic, … all kinds of approaches, diagnoses, things she typed on the computer, I did them in that period. For example, communication, listening to the patient, questions she asked … I did whatever she did. (FG-1/Part.9/Line 521–527) (G6)

Those who claimed that education for the training of ‘good physicians’ is an *invalid* and *futile effort* considered it impossible to instill the adoption of certain attitudes – because personality traits are static.

The possibility that medical education might actually *detract from professional identity development* is also worth mentioning. Besides determinants like unsuitable working conditions and unskilled/incompetent role models, other stated factors included: 1) unsupervised encounters with role models; 2) negative influences in clinical practice; and 3) the discrepancy between the theoretical, preclinical knowledge, and actual clinical practice.

When I entered the medical faculty, seems like I was confident that I was going to be a good doctor. But as a result of the things I experienced during internship in internal diseases … I became careless about patients … the resident don't care about you, nurses ignore you, orderlies ignore you; after living these situations, after all that tiredness, when I saw patients disregarded me either, I was burst into tears once asking myself whether it would have been better if I did not choose to be a doctor. (FG-3/Part.2/Line 669–680) (G6)

### The efficiency of the current medical education

Participants’ answers to the question, ‘If you had a chance, what advice would you give to the institutional administrators or bureaucrats?’ were based almost entirely on G6 student responses and reflected three distinct contexts: 1) the curriculum/program structure; 2) the teacher-student relationship; and 3) the healthcare system (Table 4). All three themes were interrelated, but some were discussed more intensely than others. One such example is ‘the excessive number of the students’ in AUFM. Students expressed their desire to be educated in small groups, in closer contact with professors and other faculty members, and to be observed and supervised by them not only during clinical terms, but also throughout the first 3 years. The fact that the students were often taught in large groups which exceeded the capacity of the facilities was stated repeatedly. Students believed this to be detrimental to the student-staff relationship, causing students to feel invisible; deprived of a positive, attitude-changing interaction with educators; and unrecognized as unique individuals. To resolve this situation, students proposed that their input be considered when designing the education program.

**Table 4:** The thematic classification of students’ opinions upon the education program currently being implemented in AUFM in the context of ‘being a good physician’

‘If you ever had a chance, what advices would you give to the institutional administrators in order for them to be able to raise good physicians?’			

		Theme/Opinion	
		
Context	Dimension	Grade-1	Grade-6
Structuring education programs/curriculum	Substructure	The impossibility of education in crowded classes	The impossibility of education in crowded classes
	Theory/practice balance	Education should be integrated. Students should be able to get in touch with patients in the early stages of education. Clinical studies should start earlier. The program should not be based on memorizing.	Education should be integrated. Students should be able to get in touch with patients in the early stages of education. Most of the education should be practically given at the bed-side. The professional skills practices should be made with patients in the first 3 years. There should be practical work in the first 3 years.
	Communication education	–	Communication education should be given at the clinics too.
	Educational objectives	Education should be in accordance with the real life.	Education should be in accordance with the real life. It should be aimed to raise general practitioners.
	Structuring education programs	Education and the educators should be shaped according to the student feedbacks. Self-studying/self-learning opportunities should be enabled.	Education and the educators should be shaped according to the student feedbacks. Self-learning skills should be taught to students. The professor-student interaction should be increased in basic medical education. Professors should be present at the educational activities at the clinic.
Educator/instructor-student relationship	The effect of role models	The educator-resident-student communication should be strengthened.	The educator-resident-student communication should be strengthened.
Healthcare system	Structural problems	–	Educators should work full-time. Pay for performance system should be abolished. The time spared for each patient should be increased. TUS should be repealed.

Students also suggested an immediate cause-and-effect relationship between observed educational shortcomings and larger healthcare policies – such as pay for performance, new faculty work regulations, changes in the referral chain that resulted in excess patient volume at university hospitals, and the Specialty Examination in Medicine^1^ (TUS). According to the students, it follows that a professor or a resident, who is supposed to see a large number of patients, cannot spare adequate time for clinical education.

Another critical issue raised was ‘the sharp contrast between the preclinical/basic medical education programs and clinical education period’ – causing these two components to be almost totally disconnected. Students seemed to recognize the importance of values, attitudes, and skills taught in the preclinical phase, but feel this material is not easily translated during clinical training. This inconsistency was apparent in several respects. First, structural dimensions, such as those mentioned above, preclude realization of the theoretical education of professional values. In addition, theoretical education programs – where professional values are discussed – rarely touch upon the problems faced within actual clinical practice. Second, the attitudes and behaviors of clinical educators do not fit the ‘good physician’ picture painted in their earlier training, and students’ ideals are eroded during the instructor-student encounters at the clinic. Yet, students still expressed desire to learn more about the profession by observing their teachers.

I wish there was communication inside the school. As we said, for instance, there are three layers; professors, residents, interns. Everyone should be informed about each other. Professors should be up to the condition of their residents, and their students, and I mean, be in cooperation with them. Everything should be done together. (FG-1/Part.8/Line 850–859) (G6)

When professors are interested, you feel more valuable there. Well, some don't see [you], you become like a ghost in front of them. When it is so, you think ‘why am I standing here?’ In the end, you are on duty too; I mean you do something too. (FG-1/Part.3/Line 770–773) (G6)

Third, students offered some tangible suggestions for remedying the disconnect between the preclinical and clinical terms – including earlier patient exposure, contextualizing more theoretical education within the existing social and healthcare systems, extending preclinical content (e.g., communication skills) into the clinical years, and ensuring that educators receive proper training.

Actually we used to take professional skills classes … we received lectures on talking to a patient, on communication. How well we had learned there. Then when we came up to the internship, we saw that they were using the term ‘kicking out’ [the patient]. Although you aren't involved in it, you try not be like that, I saw some who were affected by these expressions, and shifted in that way by imitating residents (FG-1/Part.2/Line 424–429) (G6) Recently I've told a patient ‘I understand you’. He almost laughed at me so as to say ‘how come you understand me?’ I am in triage; I separate the ones who have a state of emergency and who don't …. For instance, the child has a stomach ache for a month. I say ‘come to the out-patient clinic tomorrow’. He [his father] says ‘do we need to die in any case?’ When I say ‘OK, I understand you too but’, everything finishes there. (FG-1/Part.9/Line 487–493) (G6)

Another topic frequently addressed was the effect of practice in professional education. Although it was difficult to determine the specific knowledge, skills, or attitudes, students saw little value in a ‘theoretical’ education that could not be validated in practice.

But there is no connection of the things they do in theoretical [program] with the practice. How can they expect me to be a good physician, while I keep seeing these now? Now that we have such a thing in our own hospital, how can they expect me to make interventions when I go somewhere as a practitioner with this background? Or after I see such a physician-patient relationship? (FG-2/Part.8/Line 543–547) (G6)

## Discussion

This study represents the first qualitative inquiry into Turkish students’ perceptions of medical professionalism. Although limited to a single institution, the findings provide a basic framework of the issue in our unique cultural context. It is hoped that the results will serve as a guide to better focus future research.

As mentioned, a limitation of our study is the fact that it was performed at a single medical school (AUFM). The results would likely have been more generalizable had the data been collected from multiple institutions. However, because AUFM is among the largest medical schools in Turkey, the views elicited toward medical education policies likely reflect those of other student bodies.

Professional identity develops under the influence of many factors over time. To gain a comprehensive picture of students’ perceptions of professionalism, as many determinants as possible should be taken into consideration. In this study, students’ accounts related to ‘being a good physician’ were analyzed primarily in the context of formal medical education. However, less observable aspects of education – such as the cultural environment, quality of role models, relationships with peers and superiors, available resources and facilities, and opportunities for self-improvement and teamwork, could not be investigated in detail. For this reason, the interpretation of study findings is necessarily limited.

Last, this study is a cross-sectional inquiry, which did not allow researchers to observe how students’ perceptions may change over time. Similarly, although students are likely to act on what they believe to be true, the cross-sectional design precludes any definitive discussions of causal relationships.

Generally, freshmen (G1) medical students in Turkey exhibited positive attitudes towards professionalism – but lacked any understanding of how (or if) these attitudes might function in practice. Moreover, given their inexperience in dealing with certain or compelling situations, many felt distanced from any sense of professional identity (24). Having some type of institutionally-structured approach to professionalism in AUFM might allow students to better navigate such complex and uncertain terrain. Future research may wish to explore this possibility.

Relative to professionalism, comments associated with the parallel theme of ‘being a good person’ can be considered remarkable, even though they are not specific to the medical profession (Table 2). Notably, G1 students defined more qualifications than their G6 counterparts. In a similar study conducted with G1 students in China (25), one of the three professionalism themes was defined as ‘features related with the person’, which cultural background was suggested might play a significant role in shaping. Thus, it may be reasonable to infer that students might have certain preexisting cultural prejudices of the medical profession (26). On the other hand, the difference between G1 and G6 students’ discourses suggest that such preexisting value judgments might have been replaced (or at least challenged) by those historically characteristic of the medical profession.

‘Being a good professional’ was examined within four contexts (Table 2). The first three (professional responsibility, physician-patient relationship, and physician-physician/healthcare professional relationship) and related themes match closely with findings of previous studies (1, 5, 25, 27–29). However, physician relationships with institutions, authorities, and/or drug companies were only rarely touched upon. Indeed, nearly all themes that emerged gave the impression that ‘being a good physician’ is accomplished largely within individual-level interactions (e.g., person-person encounters or individual-centered relationships) or are subject to personal choices. In other words, students tend to define the physician as a *moral agent* determined by micro-level relationships, with little recognition of macro-level dynamics (30, 31). This finding, which suggests that the students’ perceptions of professionalism lack an important conceptual dimension, should be empirically explored in different cultural contexts.

Themes concerning the ‘responsibility of the physician as an intellectual’ were voiced only by G1 students – who attributed a certain identity and responsibility to physicians both inside and outside of clinical practice. They considered a physician's role in society to include not only ‘leadership, being a model, and being a guide’, but also ‘being able to interpret the sociopolitical events’ and ‘being knowledgeable in issues such as arts and politics’.

Surprisingly, G6 students made no such statements about this aspect of professional identity. Yet, these students should have some awareness of this, because they are required to undertake compulsory service, mostly in underdeveloped regions of the country. This demands more critical thought, but may suggest that students undergo some sort of *erosion of perception* during the 6-year-medical education period.

Almost all students used similar statements to express that ‘while giving medical care, physicians should prioritize their patients and professional responsibilities, not their financial interests’. This issue has been discussed in the literature as the ‘ontological contradiction’ between *altruism* and *self-interest* (1, 32, 33). Both student cohorts emphasized that physicians should not put their financial interests ahead of their patients’ well-being. Surprisingly, in contrast to G1 students, G6 students offered no tangible behavioral examples related to this theme – perhaps because the educational experience in AUFM implicitly creates the impression that this is the proper way that medicine should operate. This effect of the hidden curriculum causes students to view an undesirable attitude not as a *contradiction –* but a *disposition*.

According to the students, the competition among physicians ‘to steal patients’ or ‘to score a better performance point’ might lead to attitudes and behaviors unbefitting of a ‘good physician’. Only G6 students provided accounts concerning this theme, probably because they are constantly in the clinical setting, and thus, witness some poignant examples of physicians’ relationships.

Belief in ‘the stableness of the person’, and that ‘education and related processes cannot change attitudes and behaviors’ was shared by both groups of students. ‘Distrust in the education programs’ can be added to these findings. The shared notion that individual attitudes (based on professional values) cannot be effectively transmitted via education should be further explored to determine its potential generalizability beyond AUFM.

In contrast to the G1 freshmen, G6 students attributed their regression away from ‘being a good physician’ to the overwhelming nature of clinical training, ill-planned institutional interventions, and other political factors. Karnieli-Miller and her colleagues claim that the dynamics related to hidden and informal curricula might have severe effects on students (34) – supporting the notion that professional experiences teach students a great deal about professionalism, sometimes in an undesired direction (35).

Three findings related to ‘the disconnection between the preclinical and clinical years’ were critical. First, by virtue of the feedback they receive, and its impact on professional identity development, students desire constant, one-to-one relationships with faculty. For that reason, they want any structural or institutional obstacles removed. Second, students consider ‘practice’ one of the main components determining one's qualification as a professional. Toward this end, they referred to the importance of working with patients more intensely and more frequently, and of providing suitable working conditions for effective learning.

Finally, ‘the influence of role models’ was repeatedly mentioned in all groups and in almost all sessions. By observing the practitioners’ behaviors, medical students comprehend how a certain moral position is realized and learn about the various dimensions of their future professional lives (36, 37). The influence of role models is virtually continuous, even though the scope and content of its effect cannot be fully supervised (16). From students’ point of view, this process should be made visible, better defined, and formalized at an institutional level.

Students also pointed out the inconsistency between the education and real life, perhaps reinforcing the belief that professionalism cannot be developed solely via formal programs. These results are also in accordance with claims that professionalism cannot be fully realized by the informal transmission of professional values and that such intervention may mask or downplay potentially contradictory influences of the hidden curriculum (1, 5, 35, 38).

In summary, our results suggest: 1) Turkish medical students suffer from erosion of perception of professional identity, and relatedly, from impoverishment of moral repertoire; 2) the ‘hidden curriculum’ plays an important role in this apparent erosion; and 3) structural changes in Turkish healthcare may negatively influence medical students’ professional identity development.

The most apparent differences between students’ accounts at the beginning and end of their medical education were the *variability of the themes and contexts* and *positive values attributed to professional practices*. In both cases, G6 students’ expressions were more superficial than those of G1 students.

This study supports the phenomenon conceptualized as *proto-professionalism* by Hilton and Slotnick (32), whereby developing a professional identity is ‘a lengthy state in which the learner develops the skills and knowledge, and gains the experience needed to acquire professionalism’. Throughout this process, two opposite forces are in play: Attainment and attrition. The former refers to the progress of novice medical students from naïveté to practical wisdom (*phronesis*), whereas the latter refers to inclination from idealism and attachment to lack of motivation and cynicism. This dynamic manifested itself in this and earlier studies (39–41).

Given our study's cross-sectional design, the observed differences between G1 and G6 students cannot be directly attributed to their professional education in AUFM. However, several longitudinal studies examining specific changes in medical students’ professionalism (e.g., empathy, moral reasoning development, etc.) have documented this influence. For example, a meta-analysis by Neumann and associates (42) found that longitudinal studies, in particular, suggested a significant decrease in empathy during medical school. In one such longitudinal study, Lim and colleagues noted declines in medical students’ empathy between the 5th and 6th grades (43). Other studies using various research designs have shown similar declines in empathy (40, 44) and moral development (41, 45).

In nearly all related studies, the medical education experience is purported to be the primary cause of decreases in empathy and moral reasoning among trainees – usually most evident upon entering the clinical phase of medical training. Clinical experiences, inadequate mentoring, and negative role models are considered the important factors that impede professional identity development (42). Similarly, the informal and/or hidden curricula (or experiences and/or interventions attributed to them) are prominent factors as well (44).

Efficient learning strategies, supportive curricular design, reflective communication, and positive role-modeling all play central roles in the attainment process. Conversely, less engaging pedagogical approaches or environmental demands (e.g., work overload) are examples of factors underlying attrition (32). Although students in our study believed the development of professionalism cannot be achieved via formal programs, they are likely repeatedly exposed to potentially negative ‘hidden curriculum’ effects (35) – which may be more pervasive and influential than any formal curriculum (32, 46, 47).

## Conclusion

Findings gleaned from this study suggest that medical students’ educational experiences are crucial in the professionalization process. Role models and clinical practice, in particular, were shown to be important determinants. Additionally, our analysis suggests that the marketization of healthcare services in Turkey may exert an indirect, negative impact on the development of professional identity.

Turkish students are well aware that ‘good physicians’ are self-realized through their actions; thus, education programs should be designed to include those aspects of education which promote professionalism. Various manifestations of the ubiquitous ‘hidden curriculum’ – such as broader cultural and healthcare contexts – should be further investigated in larger, multisite studies. Endeavors to improve medical education programs to train more ‘complete’ physicians would undoubtedly benefit focusing on those guiding forces which often remain implicit and unseen.

## Footnotes

1 Specialty Examination in Medicine (TUS) is a country-wide state examination, held twice a year in Turkey, for post-graduate physicians according to which they can be placed into various institutions in order to receive a medical specialty education.
